# Engaging people who use drugs in clinical research: integrating facilitated telemedicine for HCV into substance use treatment

**DOI:** 10.1186/s40900-023-00474-x

**Published:** 2023-08-02

**Authors:** Andrew H. Talal, Saliyah J. George, Lillian A. Talal, Arpan Dharia, Ana Ventuneac, Gloria Baciewicz, Ponni V. Perumalswami, Suzanne S. Dickerson

**Affiliations:** 1 Division of Gastroenterology, Hepatology, and Nutrition, Department of Medicine, University at Buffalo, State University of New York, 875 Ellicott Street, Suite 6090, Buffalo, NY 14203 USA; 2 Division of Liver Diseases, Department of Medicine, Icahn School of Medicine at Mount Sinai, New York, NY USA; 3 New York University, New York, NY USA; 4 START Treatment and Recovery Centers, Brooklyn, NY USA; 5 University of Rochester, Rochester, NY USA; 6 Division of Biobehavioral Health and Clinical Sciences, School of Nursing, University at Buffalo, Buffalo, NY USA

**Keywords:** Hepatitis C virus, Patient and public involvement, Clinical research engagement, Thematic synthesis

## Abstract

**Background:**

People who use drugs (PWUD) have difficulty participating in clinical research. We evaluated approaches to engage PWUD in clinical research, using facilitated telemedicine for hepatitis C virus (HCV) care as an example.

**Methods:**

We analyzed stakeholder interview transcripts and study-related data (i.e., progress reports, meeting minutes) from interrelated studies to understand engagement experiences at the patient, site, and organizational levels. Stakeholders include patient-participants, opioid treatment program (OTP) staff and administrators, and research team members involved in HCV management through facilitated telemedicine integrated into OTPs.

**Results:**

Three themes emerged. Initially, the research team sought understanding of the unique culture and community of each OTP (Theme 1). The team built trusting relationships through education, communication, and feedback (Theme 2). Finally, the research team enhanced collaborative care and incorporated the patients’ voice to improve health outcomes (Theme 3). Patient-participants and OTP staff endorsed the integrated HCV care approach. Engagement practices are summarized as the CREATE framework (C = culture, R = respect, E = educate, A = advantage, T = trust, E = endorse).

**Conclusions:**

PWUD engagement in clinical research is maximized by building trusting relationships with open communication channels. Understanding the community, demonstrating respect, and augmenting knowledge are foundational for engaging PWUD in clinical research. These practices are transferable to engagement of PWUD in clinical research broadly.

## Abstract (plain language summary)

People who use drugs rarely join clinical research studies for many reasons including mistrust of researchers and lack of access to healthcare. Their joining, however, is critical to understand how to address issues affecting their communities. For ten years, we have studied telemedicine (doctor visit through a computer) to increase healthcare access for people who use drugs with hepatitis C virus (HCV). HCV infection occurs commonly in people who use drugs and is curable in almost everyone who takes treatment. We place HCV treatment through facilitated telemedicine into drug treatment programs. A case manager who is familiar to patients oversees the telemedicine encounter with the doctor. We developed themes from interviews with patients, staff, and other involved people as well as from study documents. As a first step, researchers need to understand the culture and community of the drug treatment program. Knowing the culture permits researchers to connect the goals of the study with those of the drug treatment program. It also helps researchers build trust with the program staff. We have seen that trust between the researchers and the staff in the drug treatment program permits individuals with different jobs to work together to deliver HCV treatment resulting in a cure. During the entire process, a patient advisory committee made sure the patients were partners in the research. Based upon these results, we have developed a new approach, CREATE (C = Culture, R = respect, E = educate, A = advantage, T = trust, E = endorse), that explains each step in the process.

## Introduction

Engagement in research can be defined as the meaningful involvement and partnership of study participants and stakeholders throughout the entire research process, from study design to dissemination of findings [1–3]. Engagement in research by a wide range of stakeholders can lead to more meaningful transferability and generalizability of findings and can support dissemination and implementation. Researchers should strive to ensure equitable distribution of participants and apply enrollment and retention methods uniformly without bias [1]. Data are limited, however, on the systematic study of engagement techniques, especially those targeted to underserved populations [2, 3].

People who use drugs (PWUD) often have concomitant chronic hepatitis C virus (HCV) infection due to shared infection routes. HCV treatment has been revolutionized by the advent of direct-acting antivirals (DAAs), highly efficacious treatment with minimal adverse effects prescribed for 2–3 months. Since 2014, DAAs have improved HCV treatment from low efficacy, high toxicity, which existed previously when interferon was the foundation of HCV treatment, to a high efficacy, low toxicity therapeutic regimen. HCV-infected PWUD, however, may eschew engaging in HCV management for a variety of reasons including concern for the high cost of care, which is actually covered by insurance, competing priorities, and stigma encountered in conventional healthcare settings [4, 5]. To address these issues, researchers have investigated whether facilitated telemedicine can promote access to DAAs among PWUD in treatment for opioid use disorder (OUD). Facilitated telemedicine is defined as bidirectional videoconferencing between patients and providers, aided by staff (i.e., case manager) and onsite support services, which may include phlebotomy and HCV medication dispensing. Through facilitated telemedicine, HCV care is integrated into opioid treatment programs (OTPs), which are generally considered safe, comfortable, and destigmatizing spaces by PWUD [6]. Thus, a major advantage of facilitated telemedicine integrated into OTPs is that the entire healthcare encounter occurs within a trusted and familiar environment contemporaneously with obtaining medications for OUD.

Facilitated telemedicine for HCV care is used as an example to understand PWUD engagement in clinical research. Early on in the protocol development, investigators conducted a meta-analysis that found that substance use treatment and support services together resulted in higher HCV treatment completion among PWUD [7]. In an HCV intervention study, the presence of a familiar physician in the OTP and hepatology clinic augmented PWUD participation in an HCV evaluation [8]. These observations indicated that PWUD trust of healthcare providers is transferrable between sites and provided a foundation for our protocol to assess the acceptability of facilitated telemedicine for HCV care integrated into OTPs [9]. A survey of PWUD in OTPs confirmed that 78% would be willing to receive HCV education and treatment [10]. The authors determined that a lack of HCV-related knowledge, including an understanding of the severity of the infection, was a major reason cited for unwillingness to pursue HCV treatment.

### Patient-participant and public involvement in clinical research

The RCT utilized the stepped-wedge design and was conducted at 12 OTPs in New York State (NYS). Active collaboration with the Office of Addiction Services and Supports (OASAS), the state agency that oversees a network of OUD treatment centers in NYS, facilitated the implementation of the RCT. To maintain patient-centeredness, the team developed a Patient Advisory Committee (PAC) to represent the patient-participants’ voices. The PAC consisted of 2 members from each of the 12 investigative sites and met quarterly virtually via Zoom during the period of RCT enrollment, retention, and follow-up. The research team also developed a Steering Committee consisting of the principal investigators from each of the 12 investigative sites. The Steering Committee was primarily responsible for the implementation and conduct of the RCT as it sought to ensure standardization of procedures across the different sites and to address issues as they arose. The research team also developed a Sustainability Committee that was comprised of stakeholders with an interest in sustaining and scaling facilitated telemedicine for HCV. The Sustainability Committee was comprised of representatives from government agencies, pharmaceutical and diagnostic companies, academic institutions, and nonprofit organizations and provided advice on how to sustain the intervention primarily during the follow-up period and at the conclusion of the study. The Steering Committee functioned at a partnership level in which members had decision-making authority over study procedures and implementation approaches. In contrast, the Sustainability Committee functioned at a consultation level, meaning that members provided important insights and perspectives on topics selected by the research team [11].

Critical knowledge gaps exist in the systematic study of effective engagement methods, particularly as applied to inclusion of underrepresented populations in clinical research. We sought to understand PWUD engagement in clinical research. In this context, engagement refers to recruitment, enrollment, and retention of PWUD in clinical research as well as their active membership on the research team to guide study design and study implementation. PWUD difficulty in accessing DAAs provides an excellent foundation upon which to study engagement of an underserved population in clinical research. Expanding PWUD access to DAAs necessitates an initial understanding of barriers and facilitators to pursuit of HCV treatment and requires subsequent formulation of specific approaches to engage PWUD in clinical research. Our research team evaluated data from multiple stakeholder studies (patients, administrators, staff, and research team members) who provided input on the study design, implementation and dissemination to understand and offer solutions for restricted HCV care access among PWUD [12]. From the data in these studies, we utilized hermeneutic phenomenology to understand engagement practices by stakeholders in HCV care in the OTP setting that can be transferable to other settings targeted to PWUD.

## Methods

Based upon stakeholders and patient-participants’ experiences of clinical research participation, the team conducted a secondary analysis to synthesize data across several interrelated studies to address the following question: What approaches promoted engagement of PWUD in clinical research? The goal was to understand PWUD engagement in clinical research from the experiences of patient-participants and other stakeholders as collaborators contributing as research team members. The team performed a thematic analysis to integrate data derived from primary and secondary sources to address engagement practices.

### Data sources

Prior work of facilitated telemedicine for HCV care included our pilot study [13] and subsequent RCT [12], conducted at 12 OTPs in New York State. Briefly, an inclusion criterion of the RCT was active insurance that provided DAAs at no cost. The RCT was designed consistent with the Multidimensional Framework for Patient and Family Engagement in Health and Health Care [11] for patient-level assessments. We used the principles of community-based participatory research (CBPR) [14] for organization-level assessments. A description of data sources is presented in Table 1.

**Table 1:** Description of data sources

Data sources	Description
Case Manager	Recruitment strategies and notable cases are described in a recent publication [15]
Patient-Participants	A pilot study to assess the feasibility of telemedicine conducted in OTPs [13, 23] Publication comparing study patient-participant satisfaction with telemedicine compared to in-person evaluations from 25 patient interviews [29] Publication evaluating patient-participant challenges and benefits of HCV treatment from 25 patient interviews (manuscript submitted)
Patient Advisory Committee	Representation of the patient voice throughout the study Information about the PAC derived from conference presentation by the PAC Chair and member Meeting minutes and agendas
OTP Staff-Participants	OTP staff experiences with facilitated telemedicine from 45 staff interviews Staff experience addressing facilitated telemedicine integrated into the OTP (manuscript submitted) Administrators describing factors required for telemedicine sustainability) [17]
Study-Specific Documents:	Progress reports and meeting minutes

*OTP* opioid treatment program, *HCV* hepatitis C virus, *PAC* patient advisory committee

### Thematic analysis

The team analyzed textual data from several sources (Table 1) using hermeneutic phenomenology [15, 16] and consistent with prior similar studies [15, 17]. The goal of hermeneutic phenomenology is to understand situations and interactions as they were experienced by individual stakeholders within a context of time, place, and situational influences. In hermeneutic phenomenology, stakeholders’ engagement experiences, as revealed through interview transcripts and contemporaneous study-related documents, provide the analytical data. The analytical goal is to interpret common meanings and shared practices of participants’ experiences.

We used the hermeneutic phenomenological approach to understand the experiences of engaging PWUD in clinical research. These interviews, study-related data, and presentations are the basis of understanding of the past, present, and future possibilities that forms the horizon of understanding informing taken-for-granted meanings. The language of the participants and other sources are the data for analysis of the meaning of the experience in context that is explicated. The understanding of participants’ experiences is also useful for future transferability to similar contexts, such as engagement of PWUD in other types of research studies. We chose the approach of hermeneutic phenomenology, seeking temporal meaning and situational context, over, for example, grounded theory, which focuses on basic social processes and theory development. Similarly, we chose the hermeneutic phenomenology approach, as opposed to the psychology-informed interpretative phenomenological analysis (IPA), which focuses on the construct of cognition [18–21].

The five-member analysis team included a Professor of Nursing who is a hermeneutics expert, a Vice-President for Research and Evaluation at an OTP consortium, 2 case managers (CM), and a physician-scientist with expertise in viral hepatitis. Guided by the hermeneutics expert, the team interpreted the texts in a reflective process following iterative steps documented in weekly analytical meeting notes [16]. The team maintained rigor throughout the interpretative process by applying de Witt and Ploeg’s framework [22]. Additional coauthors provided input on the thematic interpretations. The team achieved openness through the continuous, systematic process of auditing the team’s interpretations. The team achieved concreteness and resonance by using quotations that illustrate participant examples depicting the themes. Based on the analytical interpretations, the team reflected on the development of practical advice and interventions designed to promote engagement of underserved populations in clinical research. Once developed, the team examined the themes and constitutive pattern, which links the themes together, for coherence and comprehensiveness until saturation and consensus amongst all team members was achieved.

## Results

Review of qualitative data of engagement experiences of stakeholders revealed three main themes and one constitutive pattern to explicate “Engaging PWUD in Clinical Research”.

### Theme 1: Understanding the culture and community of opioid treatment programs

When entering a new venue, such as an OTP, the research team including the CMs and staff, strove to understand the site’s culture and community. Individual CMs, assigned to each study site, were responsible for all study procedures, and represented the study leadership. In the RCT, CMs initially understood the compendium of competing priorities facing PWUD, their daily struggles, and their substance use experiences.

“*The shaming and the guilt is huge in addiction. It has to do with the stigma of addiction. A lot of us in our community… We have this poverty of knowledge about addiction*” (Administrator).

PWUD are often stigmatized, which thwarts accessing quality care in conventional healthcare settings. PWUD often “*try to hide [their drug use] from some people [including staff] because they don't want to be viewed as unworthy of care”* (Staff). OTPs promote patient-centeredness that support PWUD as they treat OUD in a community-like, destigmatizing environment. “*It seems everybody really cares, and it’s personal with a lot of people. We're not just numbers"* (Patient-participant).

*“The team working in the OTP are all very passionate people with empathy and non-judgmental approaches towards individuals who have substance use disorders. They do a really nice job making patients feel welcome and respected… We frequently hear that they encounter and deal with [offsite] healthcare providers’ judgement [and] stigma. That is also what makes [the OTP] successful when you have patients that trust you, feel respected, and are treated like a normal human being and not judged based on the fact that they are somebody with a substance use disorder… Patients have said that in other clinics you feel like you're a number, whereas in our clinic we have heard many times patients feel like ‘I am somebody, they know my name, they treat me well’”* (Administrator).

A staff member described an example of PWUDs’ everyday challenges, “[Patients say] ‘*it is very difficult to go to a new office because of the stigma and maltreatment… The OTP is great. I’m comfortable here’”*.

**Subtheme 1a: Laying the groundwork and aligning OTP values with the research objectives.**

Prior to implementing the RCT, a pilot study enabled investigation of the feasibility and acceptability of telemedicine integrated into OTPs as well as assessment of workflows, capacity, social environment, and stakeholder needs [13, 23]. For example, sites lacking sufficient broadband capacity were provided with appropriate infrastructure for connectivity capabilities. An OTP administrator explained:

*“A number of years ago, we integrated primary care into an OTP, and it failed. We failed because we didn't do what the [RCT] project did right… We did not do the groundwork… Developing the relationships and listening to what the patients actually needed*… *If you try to impose a structure without understanding the culture, it's not going to work. [The research team] really listened and took the time to understand the culture and then worked within that culture*.”

A patient-participant shared their initial skepticism about clinical research and how their feelings changed once they initiated HCV treatment:

“At first, I was like, ‘that’s [study] full of sh**.’ They always say things to get you to join, but I’m glad I did. I knew that I had hep C, and I thought that I was going to die from it because I didn’t want to get the treatment... After [a peer] told me about it, I came... I feel great now, no side effects. I called [DAA therapy] the wonder pill”.

To address misconceptions about HCV treatment, the research team and OTP staff collaborated to educate patients and staff about HCV management including modern HCV therapy (i.e., DAAs) and the concept of telemedicine [10, 24]. As one patient-participant remarked, “*Who could have believed that you could talk to someone over the telescreen to give you information about a pill that could help save your life?”.*

One staff-participant remarked about the informative experiences through the pilot study:

*“When we did the pilot study, there were a number of things that were new, but with the randomized study, a lot of the things that we learned in the pilot study were improved on. This study was a win-win for not just us as staff, but also for patients*”.

OTPs promote patient-centered, harm reduction approaches [25]. The research team strategically implemented patient-centered care that was “*a one-stop shop of health care delivery*” while simultaneously building relationships with OTP staff.

The pilot study synergized alignment between the study objectives with those of the OTP and the state entity that oversees substance use treatment (i.e., OASAS) to gain buy-in from all stakeholders. The facilitated telemedicine model that was developed during the pilot study, by ensuring HCV screening and treatment delivery with a patient-centered focus, accomplished the goals of the OTP and OASAS. Understanding the culture and laying the groundwork are prerequisites for effective implementation of a rigorous clinical research study.

### Theme 2: Building trusting relationships through education, communication, and feedback

The investigators utilized a variety of approaches to build trusting relationships between the research team, OTP staff, and patient-participants to promote engagement in clinical research. Up-to-date education about HCV formed a bedrock upon which engagement was built. An administrator reflected:

*“Before we ever even started enrolling patients, they [research team] provided education to all of our staff on HCV… It was really helpful for our staff to understand HCV and to know about treatment options because that has changed so much in the last few years. Staff [are] able to have conversations with their patients [about HCV]… No longer being treated for six months. [It] was really important to have up-to-date information about the disease [and] disease management”.*

Essential knowledge components included updates on recent HCV therapeutic advances, especially the development of DAAs. A CM noted:

*“[Patient-participants] thought that the therapy was interferon… Patients had to be educated on the reasons why they were being treated, CMs had to familiarize patients with the [study] workflow, and the clinical staff or the HCV provider answered all questions*.”

Misinformation regarding HCV therapy was a barrier to engagement that was overcome by providing education to staff and patient-participants [10, 24, 26]. By equipping OTP staff with HCV knowledge, they became force multipliers in delivering HCV education to patient-participants and guiding them through the care cascade.

*“Patient education [helps patients] to understand more. Some of them are afraid that we’re still using interferon… We had to educate them and explain that we’re not using that now and that [DAAs have] minimal side effects” (Case manager).*

Prior to improved HCV knowledge, many patient-participants considered HCV a lifelong infection and, in some cases, a death sentence. *“As you can imagine, having hep C and being cured is a really big deal for patients”* (Staff). An HCV cure opened opportunities to improve overall health and well-being. “*Getting cured from hepatitis C prolonged my life*” (Patient-participant). Another patient-participant acknowledged the lack of transmission potential resulting from a cure, “*[I] definitely feel better, more confident. Before, I had to worry if my wife picked up my toothbrush…[or] my son. It [cure] was a weight off my shoulders”*.

The focus on education as a method to engage patients and to assuage anxiety related to novel interventions extended to facilitated telemedicine, as a staff member emphasized, “*Education is the most important part. You have to stress that [telemedicine] is new. It’s important to let them know that it is HIPAA-compliant [and] confidential*”.

Annual onsite staff appreciation and learning lunches were a particularly effective approach to deliver HCV education and engage the entire OTP staff and patient-participants. The lunches also permitted opportunities to discuss study procedures and obtain real-time feedback to address challenges. Through these activities, the research team became active participants in OTP workflows and activities.

*“[When the research team visited], they gave us lunch and explained more about the project. [It] was very helpful to see who was running this [study]… [It] gave us an opportunity to ask questions”* (Staff).

These events also promoted OTP staff endorsement as an administrator noted, “*[Lunches] were done at very appropriate and frequent intervals so that staff could course-correct if we needed to*”. At one event, patient-participants in their capacity as research implementation partners expressed concern over loss of confidentiality around an HCV diagnosis if others observed co-administration of methadone and DAAs. To maintain privacy, OTP leadership had dividers installed surrounding the dispensing window. Thus, education, communication, and honest feedback served as the foundation of trusting and respectful relationships.

### Theme 3: Enhancing collaborative care and incorporating the patient voice to improve health outcomes

An engaging care team involved collaboration between study supported CMs and OTP staff consisting of counselors, clinicians, and nurses to collectively engage patients. All team members realized the value of an HCV cure that was now available for their patients, and they worked collaboratively to engage patient-participants to achieve the desired outcome. It was clear that the teams actively promoted a consolidated effort to provide an HCV cure to their patients. The approaches used demonstrated interdisciplinary collaboration and supplementation of responsibilities to ultimately promote teamwork and improved understanding and implementation of study-related activities.

For example, the study equipped counselors to educate patients about HCV. Counselors then encouraged their patients to pursue HCV treatment. In many cases, counselors were initially responsible for identifying patients who might benefit from HCV care.

“*Counselors were the go-to [person] if patients were not available. CMs contacted us and made sure that we could get to the patient. The inclusiveness of us being there to help the patients understand [information] about their medications… It brought everything together and made it more comfortable for our patients to handle the treatment, to ask questions, and to talk to people who they already knew”* (Staff).

Patient entry into HCV treatment was facilitated by a warm handoff between the counselor and the CM, which enabled transfer of trust from the “familiar face” of the counselor to the unacquainted face of the CM. “*You need to have that trust that things are done for a reason*” (Staff). Once trust was established with the CM, patient-participants were more likely to enroll and adhere to HCV treatment. The concept of facilitated telemedicine sought to maximize patient-participant trust by integrating HCV care into the convenient and familiar environment of the OTP. In the facilitated telemedicine model, clinical staff and a CM, who were familiar to the patient-participants, were present during the telemedicine encounter and were an important component of patient engagement.

“*The idea of having the NP or PA in the room, while it [telemedicine] was going on, which was the familiar face, so it wasn't just some stranger on a screen. There was someone there who knew them and about their situation*” (Staff).

An OTP administrator further elaborated on the value of facilitated telemedicine encounters. “*If a question was asked, and the patient wasn't sure how to answer, he/she [medical person] could assist in the response… having a medical person in the room was very effective”*.

Nurses promoted patient participation by demonstrating respect for patient-participant confidentiality and privacy, which were important to some individuals, as one CM remarked:

*“Patients were more concerned [with] getting discovered, so we [had] to try different methods. Patients would prefer to take the medication away from the dispensing window. I needed to reassure them that (others) weren't going to find out*”.

Simultaneously, however, the research team acknowledged nurses’ workloads and their value in addressing patient concerns. “*The nurses were providing additional medication to the patients, so it was just a matter of adjusting to the few additional responsibilities*” (Administrator).

CMs, as one key local ambassador of the research team integrated into OTPs, ultimately facilitated patient engagement and upheld study principles. The success of the CMs’ endeavors depended upon their ability to fully integrate into the OTP workflow as team members. An administrator remarked,

“*The [CM’s role] is important because it allows the counselors to focus on the substance use disorder and it allows the social workers to deal with the mental health issues”.*

For example, CM collaboration with counselors was integral to patient-participant engagement:

“*It was a team effort from everybody in the clinic. I know that [CMs] were able to connect with the counselors to educate patients, to break down things in the simplest form. With patients, when they don't understand something, it becomes a riddle, so to have the support of the counselor and the researcher, really does educate the patient*” (Administrator).

Collaboration between the research team, OTP staff, and patient-participants demonstrated respect and trust among all participants, promoting adherence to the HCV treatment protocol and the advantage of integrated care, ultimately leading to an HCV cure. *“It’s a one-stop-shop. You stop in and get your methadone, why not stop in and get your HCV medications?”* (Patient-participant). As patient-participants were cured, the peer pipeline, combined with positive patient benefits observed by OTP staff, created a ‘feed-back loop’ reinforcing pursuit of HCV treatment for others. As one staff member indicated:

“I would endorse the process on all levels. I think it’s a beautiful thing you have. You have folks who, IV drug users, some folks… thought [HCV] was a death sentence, some thought ‘maybe, I’ll have this forever.’ Then you guys came on the scene and provided education and information and encouraged them towards treatment. I think it’s a win for everybody” .

Patient-participants also discussed the successes of achieving an HCV cure with others through “*a peer pipeline [and] spreading the word about HCV treatment to help others, anything to help mankind*.” Patient-participants also indicated that the peer pipeline was an approach to refer their colleagues for HCV treatment:

*“I think [by getting] the word out, a lot more people would do the treatment… I have five people who have reached out to me that want to get treatment.”*

**Subtheme 3a: Patient-participants as study team members**

Patient-participants were also active members of the research team. They provided initial input on the significant meaning they place on the biological outcome of the RCT, an HCV cure [27]. Patient-participants also guided RCT implementation both at the site and study levels. For example, at the conclusion of the pilot study, we created a testimonial video of patient-participant voices to understand the significance of facilitated telemedicine [23]. In fact, many of the quotes that support the CREATE framework were generated from these data. Subsequently, the video was tremendously helpful at illustrating the patient-participant perspective at site initiation visits for the RCT. On several occasions, participants from the pilot study attended these meetings in person. We also held a study-wide in-person meeting with several pilot study participants immediately prior to commencing the facilitated telemedicine intervention in the RCT. Patient-participants had decision-making input over study procedures and guided their implementation.

As the RCT proceeded, the PAC became an increasingly essential component to patient-participant engagement as defined by the PAC leader:

“*The PAC, also known as a Community Advisory Board, creates opportunities for patients receiving care at their respective clinics to provide feedback to the overall healthcare system that they attend*” (Conference presentation).

The PAC promoted collaboration amongst the research team, OTP staff, and patient-participants and sought to improve the overall patient experience. The PAC created “*a space for patients to provide input*” and to share best practices to maintain a high level of patient satisfaction with the study objectives. As a CM remarked, *“[We] would have breakfast for the patients and staff, and we would stand and introduce ourselves [during PAC meetings].*”

The PAC provided feedback on PWUD engagement practices, such as maintaining confidentiality during medication dispensing, limiting medical jargon, and compensating participants to improve recruitment, enrollment, and retention. The PAC also recommended educational materials in the form of a telemedicine introductory video and an HCV fact sheet, both of which were subsequently produced. PAC members endorsed the study’s benefits from the initiation of the study, and the PAC allowed patient-participants to share their insights, narratives, and lived experiences through a peer pipeline. Through the PAC members’ identification of knowledge gaps and recommendations of corrective actions, the PAC leader and CMs subsequently developed “*HCV educational sessions and trainings on HCV medication adherence.*”

During the COVID-19 pandemic, the PAC was fundamental in maintaining patient-participant engagement and retention while simultaneously disseminating pandemic-related knowledge. PAC meetings occurred weekly instead of quarterly. “*During COVID, the PAC met weekly virtually and aspired to provide a vehicle to promote communication, a sense of community, engagement, and retention.*” The PAC attempted to mitigate feelings of loneliness and pandemic-related stress amongst patient-participants, while promoting harm reduction. Education and study updates by text, phone, or email during the COVID lockdown reassured participants that their engagement in research remained important. It also addressed pandemic-induced changes to study procedures and reinforced the importance of continued patient-participant study involvement.

### Constitutive pattern (i.e., linking all themes together): Engaging PWUD in clinical research

**Fig. 1:**
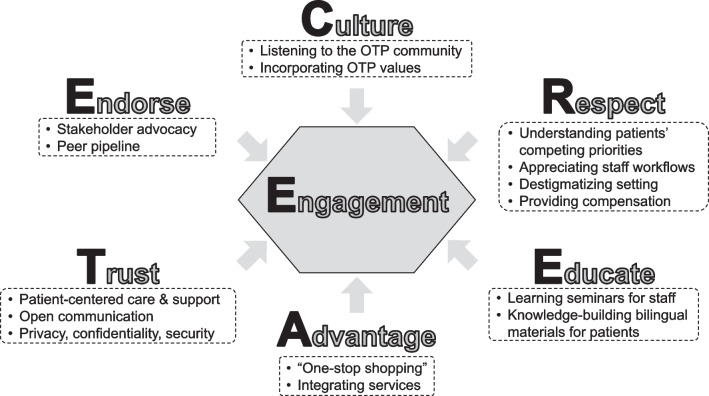
Caption: [CREATE-Culture, Respect, Educate, Advantage Trust, Endorse]. Notes: Experiences engaging an underserved population in a rigorous clinical trial led to the development of the “CREATE-Culture, Respect, Educate, Advantage Trust, Endorse,” framework to describe the process of engaging people who use drugs into clinical research. The initial step necessitates laying the groundwork through listening to the community and understanding its culture. Acknowledging and accepting the patient-participants’ unique lifestyles demonstrates respect. Researchers identified the need to educate both patient-participants and staff about hepatitis C virus (HCV). Equipping opioid treatment program staff with HCV-related knowledge enables them to confidently speak about HCV care to their patients. Patient-participants readily appreciate the advantage of facilitated telemedicine compared to offsite referral. The presence of the familiar faces of the onsite clinician and the case manager during telemedicine encounters demonstrate trust by providing patient-centered care. Dissemination of the positive benefits of facilitated telemedicine through the peer pipeline and staff appreciation of the value of an HCV cure promoted patients to endorse facilitated telemedicine and more broadly, clinical research. Abbreviation: OTP; opioid treatment program

When engaging underserved populations in clinical research, the initial step is understanding the unique culture of each OTP community. Respect for patient-participants was demonstrated through the nonjudgmental and destigmatizing setting of the OTP as well as through alignment between OTP and study objectives (Theme 1: Understanding the culture and community of each OTP). An equally important step was to educate OTP staff and patient-participants about HCV. Equipping OTP staff with HCV knowledge gave them confidence when speaking to patient-participants. Frequent staff appreciation and learning lunches improved communication and enabled course correction of study workflows. These actions opened lines of communication and feedback and fostered trusting relationships. (Theme 2: Building trusting relationships through education, communication, and feedback). Telemedicine encounters that occurred within the OTP, facilitated by CMs, enabled patient-participants to readily appreciate its advantage over offsite referral for HCV treatment. The presence of the onsite clinician provided a “familiar face” to patient-participants, thereby promoting trust. Similarly, OTP staff realized the value of an HCV cure for their patient-participants. Including patient-participants as active research team members provided opportunities for their input on study design and implementation. The PAC ensured that patient-participants’ voices were represented throughout the research and served as a key mechanism for shared leadership. Ultimately, engaged OTP staff and patient-participants endorsed integrated HCV care delivery through facilitated telemedicine (Theme 3: Enhancing collaborative care and incorporating the patient voice to improve health outcomes). The lessons learned and practical advice are derived from interactions of multiple entities pursuing PWUD engagement in clinical research (Table 2). Furthermore, our approaches can guide the design and conduct of clinical research studies and are exemplified by the CREATE framework (C = Culture, R = respect, E = educate, A = advantage, T = trust, E = endorse) (Fig. 1). Table 3 depicts several quotations supporting the CREATE framework.

**Table 2:** Engagement activities and tactics stratified by themes

Engagement activities	Descriptions
Theme 1: Understanding the unique culture and community of each OTP	
Learning the culture of the OTP	Research team learns about the OTP and its mission CM supports the OTP staff by integrating into OTP workflows
Attending OTP events	CM illustrates interest in patient-participants as individuals beyond the study CM integrates into overall OTP activities
Theme 2: Building trusting relationships through education, communication, and feedback	
Staff appreciation and learning lunches	Research team presents study procedures to OTP staff Research team educates OTP staff about HCV Research team obtains feedback from OTP staff and patient-participants to troubleshoot challenges OTP staff and patient-participants appreciate and express the value of an HCV cure
TM introductory video	CM introduces concept of facilitated TM to patient-participants
Language translations	Research team demonstrates respect and expands reach of patient-participants
Theme 3: Enhancing collaborative care and incorporating the patient voice to improve health outcomes	
Research team roles	Telemedicine provider Patient-participants connect with HCV specialist to manage HCV care remotely Case manager Facilitates TM encounters and HCV treatment Addresses patient-participant concerns and questions
Patient-participant incentives	Research team demonstrates respect for patient-participants’ time Patient-participants value financial compensation
Warm handoffs	Counselor introduces patient-participants to CM CM disseminates study-specific information CM establishes trusting relationships with patient-participants CM addresses patient-participants’ concerns or misconceptions related to study, HCV treatment, or facilitated TM
OTP staff roles	Counselor Introduces patient-participants to CM Facilitates patient-participants contact to promote recruitment, enrollment, retention, and adherence Recognizes profound changes after HCV cure in patient-participants Onsite clinician Acts as familiar face to patient-participants during TM encounters Addresses patient-participant concerns, co-morbidities, and treatment-related questions and concerns Reinforces medication adherence Enhances connectivity with TM provider Nurse Dispenses methadone and HCV medications Acts as a first line of contact for patient-participants with questions or adverse events Maintains confidentiality during dosing Illustrates trust with expanded “take-home” doses
Patient advisory committee	Represents the patients’ voices Addresses concerns about study procedures Provides feedback to research team Reinforces importance of study participation Disseminates information to patients

*CM* case manager, *HCV* hepatitis C virus, *OTP* opioid treatment program, *TM* telemedicine

**Table 3:** Quotes supporting engagement approaches (CREATE)

Abbreviation/component	Supporting quotations
C—Culture of the community	“*I would say it’s definitely [useful to have] a team approach, especially in this setting. It’s built into the setting that you need to talk to, not only the counselor, but also clinic staff. Even security guards might be involved in some aspects of what’s going on with the study… the medical director, staff, dispensing nurses too. It takes that kind of team approach to manage difficult cases”* (Case manager).
	“*We are a community amongst ourselves. We talk to one another… Thank God you [research team] came up with something that could physically help us with one of the side effects we got from our lifestyle, and you saved some of our lives. That’s what it’s about… This is who we are”* (Patient-participant).
R—Respect for patient-participants	*“We’re using patient-centered [approaches] to meet them where they are and… [help] with dynamic goal setting. We do that and patients have responded positively. We’re reducing the stigma… We’re even changing terminology that we use when we talk to our patients… For each patient, recovery means different things, success means different things, so, we’re more accommodating”* (Administrator).
E—Education regarding innovative treatment	“*There were so many stories about people [who took] interferon saying that they had such bad results from it and when the [modern HCV medications] came out, I said, ‘wow, this stuff came out in our lifetime.’ Nobody ever thought that something like this would come around in our lifetime… My whole body feels better… I hope this program continues so everybody can get the opportunity to be cured*” (Patient-participant).
A—Advantage of integrated care	*“Everybody that participated, from the doctors to the workers, I couldn't have asked for a better group. Always checking on you, wonderful group of people. [Case manager] and a few of the doctors always asking about me, and it felt great. Everybody was always concerned… It was wonderful. I still tell people to this day how excellent it was”* (Patient-participant).
	*“Combining opioid addiction therapy while curing them of their hepatitis [means] you’re achieving two goals over the long term. By treating [those conditions], you could be preventing fibrosis or hepatocellular carcinoma”* (Staff).
	*“What difference does it make as long as the information, and the treatment that he gives me, does the job? Whether he’s on the screen or whether he’s sitting in my lap, it doesn’t matter as long as he’s helping me, and my situation. The Tele… on the screen, that’s not even the issue here. The issue is that the medicine works, it’s fool-proof. Just listen to the message and get the medicine”* (Patient-participant).
T—Trust building with staff and patient-participants	“*It took a while for me to be able to gain that trust which essentially involved [the patient] sitting outside of our company and having conversations whenever [I was] available. We also had a nurse practitioner here that had a lot of experience with her. So, with those opportunities building trust, it allowed us to convince her to continue taking the [HCV] medication”* (Case manager).
	“*[The site PI] came up with the idea that [staff] are going to give patients their medication. [OTP’s staff] trust you that much to take your medication on your own. I appreciated that… By them putting that trust in me to take it on my own, that made me even strive harder to keep on taking it. It meant a lot to me that someone really trusted me to take this medication on my own”* (Patient-participant).
E—Endorsement of the intervention by staff and patient-participants	“*I think we’re all born with this innate attribute that lets us know when something is right, and when something is wrong. And the one thing about addicts is, when the good thing is out, we pass the word. And that’s what we did… we passed the word, and it was a good thing, that word that was passed. And it’s something that I hope continues for a while because there are others that still need this. It’s definitely the way to go*” (Patient-participant).

*OTP* opioid treatment program, *HCV* hepatitis C virus

## Discussion

Successful engagement of underserved populations in clinical research necessitates establishing trust within their communities so that they can be involved in the entire research process from study design through the dissemination of results. Our definition of PWUD engagement refers to both active membership in the research team to guide study design and implementation as well as improving recruitment, enrollment, and retention in clinical research. The trusted relationship between PWUD, the OTP, and its staff facilitated patient-participant enrollment and adherence to HCV treatment as exemplified in the CREATE framework (individual components underlined) [6]. The first step requires understanding the unique culture of each OTP community. Similarly, other researchers have found the OTP to be a supportive environment [28], which is consistent with recent state government guidance emphasizing patient-centered care toward PWUD [25]. Facilitated telemedicine in the OTP environment conveys respect for patient-participants [29]. CMs educate patients and OTP staff about HCV and telemedicine. The relative advantage of facilitated telemedicine compared to offsite referral promoted patient-participant trust in the intervention [23]. Our mixed method study revealed high-level patient satisfaction with facilitated telemedicine and legitimized it as a healthcare delivery modality [29]. The patient-participants extolled positive experiences about the attributes of telemedicine, which were disseminated through the OTP community via a peer pipeline [30]. Simultaneously, OTP staff valued an HCV cure for their patients. Based upon the positive messages from peers and OTP staff, patients endorsed HCV care through facilitated telemedicine and concomitantly clinical research.

Patient-participants and OTP staff described the OTP as a community defined as a group of people sharing common attitudes, interests, and goals [14]. In delivering HCV care, the research team sought to collaborate with the OTP staff to improve PWUDs’ lives. Research engagement approaches sought to build upon the community’s strengths and resources consistent with CBPR principles that recognize the community as a unit of identity [31]. These approaches also recognize equal stakeholder participation, co-leadership, and open communication channels, consistent with the Carman et al. framework [11]. The framework facilitates elucidation of the contextual factors and processes of engagement and is designed to increase clinical research participation by underrepresented populations [32]. It describes engagement activities along a continuum ranging from consultation to partnership to shared leadership. Engagement can also occur at different levels (i.e., the patient, site, or organizational levels). It also can be used to describe how OTP staff and stakeholders influence engagement and the extent to which it occurs (Table 4). In our context, annual staff appreciation and learning lunches provided opportunities for co-learning by patients and OTP staff. Shared leadership principles ensured that all stakeholders were heard, and their advice was acted upon, such as site-specific workflows [33, 34]. The alignment of stakeholder objectives increases the likelihood of intervention success.

**Table 4:** Stakeholder consultation, involvement, partnership, and shared leadership in clinical research

Levels of engagement	Stakeholders			
	Patient Advisory Committee (Clinical research participants)	Opioid Treatment Program (Patients, leadership, administrators, staff)	Research Team (Academic institutions, research staff, case managers, patients, steering committee)	New York State Government (OASAS, NYS DOH)
Patient Care	• PAC ensures that the patients’ voice is represented throughout entire research process.	• OTP staff value the positive health outcomes and endorse the study to their patients. • Patients support a peer pipeline endorsing HCV treatment to other patients.	• CMs promote trust, integrate into the OTP staff, address competing priorities, and facilitate telemedicine encounters. • Patient feedback on procedures is invaluable to the research team.	• OASAS encourages patient-centered care.
Organizational design	• PAC comments on privacy and confidentiality issues. • PAC recommended HCV education for patients. • Patients disseminate information via peer pipeline.	• OTP staff participates in HCV education via learning lunches. • OTP staff provides feedback to research team. • OTP staff integrates HCV care into OTP workflows.	• Team leadership aims to understand the culture and community of each OTP. • Team leadership incorporates OTP values into the study objectives.	• OASAS facilitates OTP engagement by connecting study and OTP leadership. • OASAS approves HCV care in study OTPs.
Policy	• PAC disseminates study outcomes.	• OTP leadership aims to sustain onsite HCV care.	• Team leadership disseminates the study outcomes to OASAS, NYS DOH, and beyond.	• OASAS recommends HCV testing and treatment in all OTPs. • DOH funds telemedicine for mental health, substance use, and HCV.

Additional study stakeholders include specialty pharmacies, pharmaceutical and diagnostic companies and videoconferencing companies. They were responsible for establishing protocols in their respective areas. They also advised on deployment of telemedicine for HCV outside of New York State as well as telemedicine expansion for other health conditions

*CM* case manager, *HCV* hepatitis C virus, *NYS DOH* New York State Department of Health, *OASAS* Office of Addiction Services and Supports, *OTP* opioid treatment program, *PAC* patient advisory committee

An initial motivation promoting study enrollment is the belief of personal benefit [2]. HCV-infected PWUD, however, rarely appreciate the severity of HCV infection [35]. Therefore, HCV education is foundational for patient-participant engagement in HCV care. Similarly, a knowledgeable OTP staff supports patients under HCV care through frequent counseling [36]. CMs worked collaboratively with the interdisciplinary OTP staff to facilitate study enrollment. Counselors educated patients and facilitated initial interactions as well as study retention. Nurses dispensed DAAs, and site clinicians managed adverse effects. Situating telemedicine encounters in trusted environments promoted patient-participant engagement. These approaches simultaneously build infrastructure to support meaningful engagement and promote strong stakeholder relationships [3].

Virtual technology, utilized through facilitated telemedicine and the PAC, promoted PWUD engagement in clinical research. Telemedicine encounters permitted frequent patient-participant-provider interactions that promoted high-level patient satisfaction [23, 29]. Additionally, PAC members ensured representation of patient-participants’ voices. Because many patient-participants had pre-pandemic experience with virtual technology, they swiftly embraced it for communication during the lockdown. Similarly, sites readily acclimated to telemedicine during the lockdown based upon prior experiences. Virtual visits have facilitated interactions with other underserved populations, such as HIV-infected women from underserved neighborhoods. Virtual interactions became indispensable during the pandemic to minimize potential COVID-19 exposure, and they expanded the repertoire of recruitment and enrollment strategies while maintaining flexibility, such as through text, phone, and email.

In the RCT, a variety of stakeholders provided input at various stages of the research at the patient, organizational, and policy levels (Table 4). These interventions were tested not only as part of an RCT, but during the natural experiment of the COVID-19 pandemic. NYS government through various sectors has realized the value of telemedicine both through the development of a telehealth implementation toolkit [37] as well as through a NYS health commissioner’s determination [38] to permit continued use of telemedicine for prescription of OUD treatment.

The results presented here inform engaging underserved populations in clinical research. They were derived from only one state at state government-regulated sites, which may impact transferability. OTPs in New York State are under the jurisdiction of OASAS and their guidance, which emphasizes patient-centered care, may not be transferable to other states. We assessed engagement practices related to facilitated telemedicine conducted as a clinical research endeavor, which may limit transferability to clinical care encounters. Another limitation relates to an RCT inclusion requirement of at least six months enrollment in the OTP. Whether the same engagement approaches would be successful with individuals not enrolled in OUD treatment is an open research question. The team assessed engagement practices qualitatively based upon stakeholder’s experiences with the intervention. Another limitation is that PAC members were not engaged as part of the analysis team, although an early draft of the manuscript was member-checked by the PAC leader.

## Conclusion

A repertoire of approaches is needed to engage underserved populations in clinical research. The initial step is listening to and understanding the unique OTP culture and demonstrating respect for all stakeholders. Facilitated telemedicine aligned with the OTP mission as existing OTP workflows support patient-centered healthcare delivery [25]. Patient-participants readily identified the competitive advantages of onsite telemedicine, and OTP staff appreciated the value of an HCV cure. Future research should investigate the transferability of the components of the CREATE framework.

## Data Availability

The datasets used and analyzed during the current study are available from the corresponding author on reasonable request.

## Abbreviations

CBPR: Community-based participatory research
CM: Case manager
DAAs: Direct acting antivirals
HCV: Hepatitis C virus
HIPAA: Health Insurance Portability and Accountability Act
NYS: New York State
OASAS: Office of Addiction Services and Supports
OTP: Opioid treatment program
OUD: Opioid use disorder
PAC: Patient advisory committee
PCORI: Patient-Centered Outcomes Research Institute
PWUD: People who use drugs
